# A Cross-Sectional Study Demonstrating a High Prevalence of Skin Rash to Diabetes Medical Devices: An Underestimated Problem

**DOI:** 10.1177/19322968251336261

**Published:** 2025-05-07

**Authors:** Josefin Ulriksdotter, Thanisorn Sukakul, Magnus Bruze, Nils Hamnerius, Martin Mowitz, Cecilia Svedman

**Affiliations:** 1 Department of Occupational and Environmental Dermatology, Lund University, Skåne University Hospital, Malmö, Sweden

**Keywords:** contact dermatitis, continuous glucose monitoring, insulin infusion systems, medical device, type 1 diabetes

## Abstract

**Background:**

Adverse skin reactions to continuous glucose monitors (CGMs) and devices for continuous subcutaneous insulin infusions (CSIIs) (“diabetes medical devices” (MDs)) are well known. However, epidemiological studies on prevalence and skin rash details are lacking. The objective of this study was to describe the prevalence and details of skin rash to diabetes MDs in adults with type 1 diabetes.

**Method:**

All adult individuals (≥18 years) with type 1 diabetes attending outpatient diabetes clinics at two hospitals in southern Sweden were invited to participate (n = 1943) in a questionnaire study.

**Results:**

The questionnaire was completed by 667. Of the respondents 95.1% had used CGM and 36.7% had used CSII. Skin rash to the devices was reported by 42.1% of CGM users and 44.9% of CSII users. Skin rash was reported with use of all types of diabetes MDs. For diabetes MDs with ≥50 users, 18.0% to 56.5% of the participants with skin rash had to change the device more often than recommended and 4.0% to 18.0% had to stop using the device due to skin rash. In multivariable analyses, the odds for skin rash to diabetes MDs were higher among younger individuals and individuals with childhood atopic dermatitis. Odds increased with use of higher numbers of devices. Of the participants with skin rash, 13 of the 289 (4.5%) had been investigated for contact allergy.

**Conclusions:**

Skin rash to diabetes MDs is common. The problem is underdiagnosed in clinical practice. With use of diabetes MDs expected to increase, an increasing prevalence of skin rash is to be expected.

## Introduction

Continuous glucose monitors (CGMs) and devices for continuous subcutaneous insulin infusion (CSII) (in this article defined as diabetes medical devices (MDs)) are widely used among individuals with type 1 diabetes. CSII devices are used with an infusion set or as patch pumps that adhere directly to the skin. Compared with self-monitoring of blood glucose and multiple daily injections of insulin, use of these diabetes MDs is associated with improved health-related quality of life, improved glucose control and, ideally, reduced long-term disease complications.^1-4^ However, as the diabetes MDs need to adhere closely to the skin for prolonged periods the skin adhesive must perform accordingly and many cases of severe skin reactions including allergic contact dermatitis (ACD) to the devices have been reported.^5-14^A newly published review^15^ indicates that skin reactions associated with the use of diabetes MDs are common and most likely underdiagnosed. Because of scarce epidemiological data on skin rash as a primary outcome, the prevalence and incidence of skin rash associated with the use of diabetes MDs are still uncertain.^15^ The aim of this study was to assess the self-reported prevalence and characteristics of skin rash to diabetes MDs in adults with type 1 diabetes.

## Methods

### Participants

In this cross-sectional questionnaire-based study, all adults (18 years or older) with type 1 diabetes (n = 1943) attending two outpatient diabetes clinics in Halmstad, Region Halland and Växjö, Region Kronoberg in southern Sweden were invited to participate. Written invitation for study participation was sent by post to all potential study participants (in October-November 2021 in Växjö and March-June 2022 in Halmstad). Initially, a short, written invitation was sent including a link to more detailed information. After one week, an invitation with more detailed information was sent. After a further week an identical invitation was sent as a reminder. The potential study participants were given written information on the purpose of the study and the questionnaire. They were asked to contact our department (The Department of Occupational and Environmental Dermatology, Skåne University Hospital, Malmö Sweden) if they had any questions or if additional information was needed. Individuals consenting to participate completed the questionnaire online (see below). This study was approved by the Swedish Ethical Review Authority, dnr 2020-03160.

### Questionnaire

Demographic data surveyed were age, gender, personal history of atopy (asthma, allergic rhinoconjunctivitis, and childhood atopic dermatitis [AD]), and history of previous patch testing. For different diabetes MDs, the use of devices, localized itch (only asked for patch pumps and CGMs), and details of skin rash at the application site were asked for. Finally, treatment attempts and skin rash to adhesive dressings/tapes not associated with the use of diabetes MDs were also surveyed. The validated question “Have you had childhood eczema?” was used as a proxy of childhood AD.^16^

### Statistics

IBM SPSS Statistics for Windows (version 29.0; IBM Inc., New York, USA) was used for statistical analysis. The demographics of respondents were analyzed using descriptive methods. Data for different proportions, such as gender, a history of childhood AD, and the number of respondents using one specific type of diabetes MD are reported as percentages. Age is reported as mean and standard deviation (SD). If a respondent answered “don’t know” to a question in the questionnaire, that answer was not included in the statistical analysis.

Two-sided Pearson’s chi-square test or Fisher’s exact test were used to demonstrate the associations between two or more categorical groups. Fisher’s exact test was applied when the sample size was small (1 or more expected values less than 5). *P* values for trend (linear-by-linear association) were reported when the number of diabetes MDs used was compared between groups. The relationship between having had skin rash to MDs and possible associated factors was analyzed using logistic regression. Univariable logistic regression analysis was used to estimate the crude odds ratio (OR). The factors found to be associated with a *P* value of less than .2 were further subjected to multivariable logistic analysis. In this part of the analysis, age was categorized into two groups: 18 to 49 and equal or more than 50 years. When the mean age was compared for two groups, an independent *t* test was used. A *P* value of less than .05 was deemed statistically significant.

## Results

### Respondents

In Table 1, demographic data for the respondents is shown. In total, 667 individuals (34.3%) responded to the questionnaire. The mean age of the respondents was 49.8 years (SD = 17.6). Female respondents were significantly younger than male respondents (mean age 47.78 years [SD = 17.15] vs mean age 51.60 years [SD = 17.58] for males, *P* = .009). In total, 53.0% had a history of atopy (childhood AD, allergic rhinoconjunctivitis and/or asthma) and 21.4% of childhood AD. The proportion of females was significantly higher among respondents than nonrespondents (*P* = .007) and respondents were older than nonrespondents (mean age for nonrespondents was 47.7 years [SD = 19.1], *P* = .02).

**Table 1.** Demographic Data of Respondents.

	**All respondents** (n = 667)	
**Mean age, year** ± **SD**	49.8 ± 17.6	
**Gender (n = 666** ^**a**^ **)**	N	%
Male	351	52.7
Female	315	47.3
**Childhood atopic dermatitis** ^**b**^ **(n = 598)**	128	21.4
**Asthma^b^ (n = 648)**	93	14.4
**Allergic rhinoconjunctivitis^b^ (n = 613)**	268	43.7
**History of atopy** ^**b** **,** **c**^ **(n = 540)**	286	53.0
**Family history of atopy** ^**b** **,** **c**^ **(n = 421)**	195	46.3
**Dermatitis other location than diabetes MDs** ^**b** **,** **d**^	244	36.6
**Diabetes clinic**		
Växjö	336	50.4
Halmstad	331	49.6

Abbreviations: MDs, medical devices including continuous glucose monitors and devices for continuous subcutaneous insulin infusion; n, number of respondents; SD, standard deviation.

a One respondent did not want to state gender.

b Lifetime prevalence.

c Childhood atopic dermatitis, allergic rhinoconjunctivitis and/or asthma.

d Most common locations leg (15.0%), arm (14.2%), and hand (11.8%).

### Use of Diabetes MDs

In total, 634 respondents (95.1%) had used CGM and 245 (36.7%) had used CSII (Table 2). There was no significant difference in the proportion of the respondents that used CGM and CSII in Halmstad (Region Halland) compared with Växjö (Region Kronoberg). Of the CGM and CSII users, 238 had used both CGM and CSII, 396 only CGM and seven only CSII. In total 26 respondents had used neither CGM nor CSII. In Supplementary Table 1, the proportions of respondents that had used different diabetes MDs are presented stratified by age group. The mean numbers of different models of CGMs and CSIIs used were 1.72 (SD = 0.82) and 1.34 (SD = 0.67), respectively. In Supplementary Table 2, numbers of diabetes MDs used in different subgroups of respondents are shown. In total, 97.7% of the respondents who had used diabetes MDs had used at least one device for a minimum of six months (Table 3).

**Table 2.** Lifetime Prevalence of Skin Rash to Continuous Glucose Monitoring Systems and Continuous Subcutaneous Insulin Infusion Devices.

Different groups of users	Have ever had skin rash^a^		Total number of users	*P* value
	n	%	n	
**All CGM and CSII users**	289	45.1	641	
**CGM users**	267	42.1	634	
Number of CGM used (n = 633)^b^				<.001
One CGM	78	26.8	291	
≥2 CGMs	189	55.3	342	
**CSII users**	110	44.9	245	
Number of CSII used (n = 238)^c^				.0086
One CSII	74	41.3	179	
≥2 CSIIs	36	61.0	59	

Abbreviations: n, number of respondents; CGM, continuous glucose monitor; CSII, continuous subcutaneous insulin infusion.

a Skin reactions under CGM and/or CSII.

b One participant did not state the number of CGM used.

c Seven participants did not state the numbers of CSII used.

**Table 3.** The Odds for Skin Rash to Continuous Glucose Monitors and/or Continuous Subcutaneous Insulin Infusion Devices (Diabetes MDs) for Different Subgroups of Users.

			Have ever had skin rash to diabetes MDs							
	All		Yes		No		Statistical analysis			
	n = 570^a^		n = 263		n = 307		*P* value			
Mean age, years ± SD	49.8 ± 17.5		43.8 ± 15.6		54.9 ± 17.4		<.001			
							Univariable analysis		Multivariable analysis^b^	
							*P* value	OR [95% CI]	*P* value	OR [95% CI]
	n	%	n	%	n	%				
**Age group**							<.001		<.001	
18-49 years	275	48.2	168	61.1	107	38.9		3.30 [2.34-4.67]		2.07 [1.40-3.07]
≥50 years	295	51.8	95	32.2	200	67.8		1		1
**Sex**							<.001		.18	
Male	299	52.5	118	39.5	181	60.5		1		1
Female	271	47.5	145	53.5	126	46.5		1.76 [1.27-2.46]		1.30 [0.89-1.90]
**Childhood atopic dermatitis (AD)**										
Yes	127	22.3	81	63.8	46	36.2	<.001	2.53 [1.68-3.80]	.0025	2.04 [1.29-3.25]
No	443	77.7	182	41.1	261	58.9		1		
**Diabetes clinic**							.21			
Halmstad	285	50.0	139	48.8	146	51.2		1.24 [0.89-1.72]	N/A	N/A
Växjö	285	50.0	124	43.5	161	56.5		1		
**Number of different devices used**							<.001^c^			
1	215	37.7	52	24.2	163	75.8				1
2	162	28.4	67	41.4	95	58.6			.0075	1.88 [1.18-2.98]
3	101	17.7	69	68.3	32	31.7			<.001	5.31 [3.07-9.19]
4	60	10.5	47	78.3	13	21.7			<.001	9.15 [4.50-18.60]
≥5	32	5.6	28	87.5	4	12.5			<.001	15.10 [4.94-46.18]
**Use of at least one device for minimum six months**	557	97.7	258	46.3	299	53.7	.57	1.38 [0.45-4.27]	N/A	N/A

Abbreviations: OR, odds ratio; N/A, not applicable; n, number of respondents; SD, standard deviation.

a Only participants without missing data for gender, childhood atopic dermatitis, and number of diabetes MDs used were included in the analysis.

b Factors included in the multivariable analysis: age, gender, childhood atopic dermatitis, and number of diabetes MDs used.

c *P* value for trend (linear-by-linear association).

### Prevalence of Itch and Skin Rash

In total, 110 of the 245 (44.9%) CSII users and 267 of the 634 (42.1%) CGM users had experienced localized skin rash under their devices at some point (Table 2). The lifetime prevalence of localized skin rash under the CGM was higher among CGM users that had used CSII compared with those who had not (160/238 [67.2%] vs 128/396 [32.3%], *P* < .001). In Table 4, the lifetime prevalences of itch and skin rash to different diabetes MDs are shown. For the six most commonly used MDs (MDs used by ≥50 respondents) between 23.8% and 54.7% of current users reported skin rash to at least one diabetes MD (in the case of multiple concurrent rashes, all are reported). In total, 4.5% (13/289) of those with rash to diabetes MDs had been investigated for contact allergy with patch testing.

**Table 4.** Use of, Itch, and Skin Rash to Different Continuous Glucose Monitors and Continuous Subcutaneous Insulin Infusion Devices (Diabetes MDs).

Different diabetes MDs^b^	Use of diabetes MD^a^			Itch from diabetes MD^a^			Skin rash from diabetes MD^a^		
	n	Respondents	%	n	Respondents	%	N	Respondents	%
**CGMs**									
**At least one CGM**	**634**	**667**	**95.1**	**306**	**633**	**48.3**	**267**	**634**	**42.1**
FreeStyle Libre^c^	589	633	93.0	261	584	44.7	223	584	38.2
FreeStyle Libre 2^c^	198	629	31.5	66	197	33.5	50	197	25.4
Dexcom G6^d^	94	627	15.0	50	94	53.2	39	94	41.5
Guardian sensor 3^e^	92	626	14.7	55	91	60.4	52	92	56.5
Enlite^e^	39	611	6.4	17	38	44.7	15	37	40.5
Dexcom G5^d^	32	627	5.1	18	32	56.3	11	32	34.4
Dexcom G4^d^	27	624	4.3	15	27	55.6	8	27	29.6
Guardian sensor 4^e^	13	623	2.1	7	13	53.8	5	13	38.5
Eversense^f^	5	613	0.8	2	5	40.0	2	5	40.0
A6 TouchCare^g^	1	613	0.2	0	0	0	0	1	0
**CSIIs**									
**At least one CSII**	**245**	**667**	**36.7**	Not assessed			**110**	**245**	**44.9**
**Patch pumps**									
Omnipod^h^	54	244	22.1	34	54	63.0	23	54	42.6
**CSII with infusion sets**									
MiniMed^e^	172	242	71.1	Not assessed			75	172	43.6
Tandem t: slim X2^i^	37	244	15.2				10	37	27.0
Animas insulin pump^j^	13	244	5.3				4	13	30.8
My life YpsoPump^k^	5	244	2.0				3	5	60.0

Abbreviations: CGM, continuous glucose monitor; CSII, continuous subcutaneous insulin infusion; n, number of respondents.

a Lifetime prevalence.

b For manufacturers, see footnotes.

c Abbott Diabetes Care, Witney, UK.

d Dexcom, Inc., San Diego, USA.

e Medtronic, Minneapolis, USA.

f Senseonics, Germantown, USA.

g Medtrum, Shanghai, China.

h Insulet Corporation, Acton, USA.

i Tandem Diabetes Care, San Diego, USA.

j Animas Corporation, West Chester, Pennsylvania, USA.

k Ypsomed, Burgdorf, Switzerland.

In total, skin rash was reported to 15 different diabetes MDs (Table 4). For the six most commonly used MDs (MDs used by ≥50 respondents), between 18.0% and 56.5% of users with a history of skin rash had to change the device more often than recommended due to the skin rash. Furthermore, between 4.0% and 18.0% had to stop using the devices due to skin rash.

### Factors Associated With Skin Rash

The lifetime prevalences of skin rash to CGM and CSII were significantly higher among users that had used two or more devices than those who had used one device (Table 2). For CGMs, there was also a statistically significant trend with a higher lifetime prevalence of skin rash the more CGMs that had been used (Supplementary Figure 1). In multivariable analysis, the OR for skin rash with at least one diabetes MD was significantly higher for more than one device used, for younger individuals and for individuals with a history of childhood AD (Table 3). In univariable analysis, female gender was associated with skin rash, but in multivariable analysis, this association was not found after adjustment for age, childhood AD and number of diabetes MDs used. Females were significantly younger and had used significantly more devices than males. For childhood AD, there was no significant gender difference (*P* = .125).

### Skin Rash to Other Adhesive Dressings or Tapes

Skin rash to other adhesive dressings or tapes (not used in diabetes MDs) was reported in 93 of the 258 (36.0%) of the respondents that had experienced skin rash to at least one diabetes MD and 57 of the 362 (15.7%) of the respondents that had no history of skin rash to diabetes MDs (*P* < .001). Among the 93 individuals reporting skin rash to both diabetes MDs and other adhesive dressings or tapes, 49 (52.7%) had experienced skin rash to other adhesive dressings or tapes before the skin rash to diabetes MD started, and 44 (47.3%) after.

### Treatment of the Skin Rash

**Figure 1.**
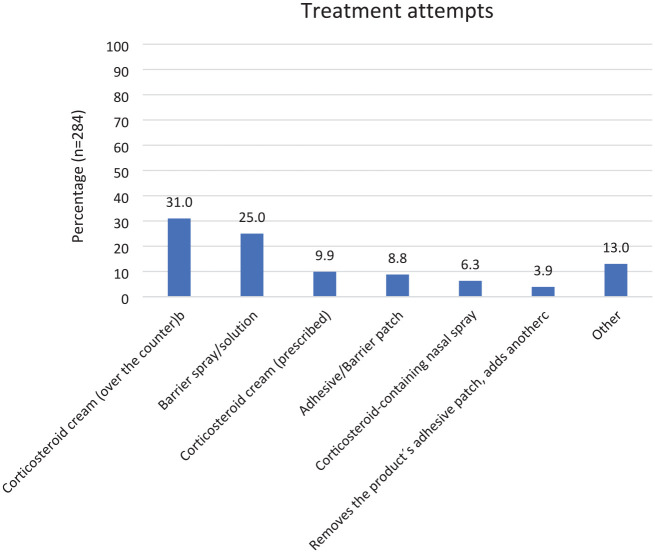
Treatment modalities used for skin rash under continuous glucose monitors and devices for continuous subcutaneous insulin infusion^a^. ^a^Multiple treatment modalities can have been used by individual respondents. ^b^Low potency corticosteroid. ^c^Removes the product’s adhesive patch and adds another type of adhesive patch.

More than half of the respondents (158/284; 55.6%) that reported skin rash to at least one diabetes MD had tried one or more treatment strategies (see Figure 1).

## Discussion

To the best of our knowledge, this is the largest study providing evidence of a high prevalence of localized itch and skin rash under diabetes MDs. Skin rash and itch were seen to a broad range of devices. The prevalences of skin rash are in line with previous studies.^13,14,17^ High prevalences of current skin reactions to diabetes MDs (34.2%-46.0%) were also reported in two studies from Denmark published in 2018-2019.^13,14^ In the same studies^13,14^ the lifetime prevalences of skin reactions were even higher than in our study. The self-reported prevalence of localized itch under the CGM was higher in one of the studies from Denmark^14^ (72.5%) than in our study. Compared with the studies from Denmark, our study included more respondents and the definition of skin reactions is more specified.

In both univariable and multivariable analyses, use of more diabetes MDs, childhood AD and younger age were associated with skin rash. The younger age group had used significantly more devices than the older age group and those with childhood AD had used significantly more devices than those without. Use of a higher number of devices could possibly reflect both the change to a new device due to skin reactions but also exposure to more potential allergens in different devices thereby increasing the risk of sensitization and elicitation of ACD. The odds for skin rash being higher among CGM users that had also used CSII supports the last-mentioned theory. It is well-known that the same allergen(s) are often found in different devices.^5-7^,^11^ This emphasizes the importance of adequate and up-to-date patch testing to ensure a correct diagnosis and advice to affected individuals on how to avoid allergen contact.

In Sweden, diabetes MDs were initially prioritized for use among children and adolescents. It is therefore not surprising that they had used a higher number of different devices. They were also potentially exposed to earlier versions of the devices possibly containing higher amounts of allergens.^18^ The range of models of diabetes MDs used differed to some degree between the older and younger participants. Different and increased exposure can therefore most likely explain the higher prevalence of skin rash in the younger age group. The use of diabetes MDs is now steadily increasing in all age groups in Sweden.^19^

Individuals with AD are more sensitive to irritant factors that might be associated with the use of diabetes MDs such as sweating, friction and irritant substances in the devices and might therefore have a higher prevalence of irritant contact dermatitis (ICD). This could possibly explain the higher prevalence of skin rash and change to new devices among individuals with childhood AD. Another possibility is that respondents having had childhood AD are more observant regarding their skin noticing skin rash at an earlier stage than those without previous eczema. Even though the results from previous studies are conflicting, individuals with AD are generally not known to have more contact allergies than individuals without AD.^20^ AD and diabetes are both common diseases and naturally having AD should not deter from use of MDs. Our data do not differentiate between ICD and ACD. One might argue that AD may indirectly increase the risk of development of contact allergy in this setting as individuals with AD who develop possible ICD or ACD may change device, potentially resulting in a vicious circle of increasing exposure to more allergens. However, the fact that patch testing was low in this group indicates that the certain diagnosis of ACD is not usually the cause for change of device.

The self-reported prevalence of childhood AD is in line with the prevalence of childhood AD (13.5% to 41.9% for the different countries) in an international study from 2021^21^ but higher than in a Swedish study^22^ from 2017 (16.3%). The validated question “Have you had childhood eczema?”^23^ has been used in several previous publications^23-29^ and has proven useful when screening for childhood AD. However, the potential risk for overestimation of the prevalence of childhood AD should be taken into consideration.^23^ The self-reported prevalence of asthma and allergic rhinoconjunctivitis was higher in the present study than in previous studies in a Swedish population.^30-32^ The association between AD and type 1 diabetes remains a subject of interest.^33-35^ While Th1 cells are considered to be mainly involved in type 1 diabetes, Th2-mediated cell response is seen in atopic diseases. With Th1 and Th2 cells being inversely related through inhibitory pathways, a lower prevalence of atopic diseases in individuals with type 1 diabetes is to be expected.^35^ However, this was not confirmed in the present study. Previous studies on the subject show conflicting results. Further studies on the relation between atopic diseases and type 1 diabetes are called for.

More than half of the participants with a history of skin rash to a diabetes MD had tried different measures to alleviate the symptoms. The most common treatments used were “over-the-counter corticosteroid cream” (low potency corticosteroid cream) and barrier sprays/solutions.

Use of barrier patches or materials under the diabetes MDs is advised by product manufacturers^36^ and is often tried among individuals with skin rash to diabetes MDs.^37^ However, it is not clear if these measures indeed protect the skin from the allergen(s). The barrier materials can contain allergens, potentially giving rise to (new) contact allergies. The treatment attempts also mean increased out-of-pocket expenses.^38^ Advice to affected individuals should be based on patch test results and diagnosis (ICD, ACD, or other).^39^ With ceased or substantially reduced contact with the culprit allergen(s) an ACD resolves. In cases with ICD, topical moisturizers and possibly topical corticosteroids can alleviate the symptoms. Skin reactions being an underdiagnosed clinical problem is supported by findings in a previous publication.^17^ Both in this study and in the previous study,^17^ the number of individuals referred to dermatologists (to be patch tested) was lower than those discontinuing use or trying nonevidence-based treatments with variable results. It is noteworthy that the proportion of study participants with skin rash to diabetes MDs that was prescribed topical corticosteroids was higher than those being referred for a dermatological evaluation (patch testing).

A substantial proportion of the participants had to change the device more often than recommended. More frequent change of the device is more time-consuming for the user and increases the treatment costs.^38^ According to a recently published article^40^ from Denmark the annual device cost for one patient using FreeStyle Libre 1, 2 and Dexcom G6 is 9713 Danish krone (DKK) (1412.3 USD), 10 054 DKK (1461.8 USD) and 13 939 DKK (2026.7 USD) respectively. According to a recently published article^41^ from Sweden the annual device cost for one patient using CGM is 12 000 to 40 000 Swedish krona (SEK) (1177.4-3924.5 USD). In Sweden almost 50 000 individuals with type 1 diabetes use CGM.^42^ Considering the high prevalence of skin rash to CGM and the high prevalence of users that had to change their CGM more often due to skin rash the increased direct costs for the extra devices are notable. Some users even had to discontinue use due to skin reactions, thereby possibly facing a higher risk of long-term diabetes complications. In a study from Italy^43^ the proportion of children/adolescents with skin rash to diabetes MDs that had to discontinue use of their devices was even higher (38.1%) than in the present study.

In Sweden diabetes MDs are widely used. According to The National Diabetes Register (NDR) in 2022^19^ 94.8% of the individuals with type 1 diabetes in Region Halland and 89.6% in Region Kronoberg used CGM and 28.8% in Halland and 40.3% in Kronoberg used CSII, which is in line with the high number of users found in this study. In Sweden, the products are publicly funded and recommended as standard of care, especially in children with type 1 diabetes. With an increased and prolonged use of these devices, it cannot be assumed that skin reactions to diabetes MDs will decrease in prevalence in the future.

Adequate primary toxicological assessments are urgently needed prior to the release of new diabetes MDs on the market. All cases of skin reactions must be reported to the relevant regulatory authorities and manufacturers, who are obliged to take precautions to prevent further cases. Prescribers must take responsibility for the initiation of investigation and management of adverse skin reactions in which dermatological assistance is needed. Collaboration between manufacturers, endocrinologists, pediatricians, dermatologists, and health care professionals involved in the procurement of these products is a necessity if proper use of the devices shall not be hampered by skin reactions.

This is a large, cross-sectional study with the assessment of skin rash as primary end point which is a strength of the study. One limitation of the study is that the time of use of the different diabetes MDs was assessed in time intervals and not as the exact time of use (which due to recall bias would have been difficult to measure adequately). The order in which the different devices had been used was not assessed and the number of users was small for some of the devices. Hence, adequate comparisons of the prevalence of skin reactions to different devices were impossible to perform. Furthermore, a higher response rate would have been preferable to decrease the risk of selection bias. It is possible that individuals with previous eczema (under diabetes MDs or childhood eczema) are more likely to participate in a study on skin reactions as compared with those without a history of eczema. Such a selection bias would lead to an overestimation of childhood AD and skin rash to diabetes MDs.

## Conclusions

Skin rash is common among users of diabetes MDs. Skin rash is seen in association with a broad range of devices. The problem is underdiagnosed in clinical practice, where affected individuals are not referred for patch testing and therefore do not receive a correct diagnosis and advice. With use of diabetes MDs only anticipated to increase, even more cases of skin reactions are to be expected.

## Supplemental Material

sj-docx-1-dst-10.1177_19322968251336261 – Supplemental material for A Cross-Sectional Study Demonstrating a High Prevalence of Skin Rash to Diabetes Medical Devices: An Underestimated Problem Supplemental material, sj-docx-1-dst-10.1177_19322968251336261 for A Cross-Sectional Study Demonstrating a High Prevalence of Skin Rash to Diabetes Medical Devices: An Underestimated Problem by Josefin Ulriksdotter, Thanisorn Sukakul, Magnus Bruze, Nils Hamnerius, Martin Mowitz and Cecilia Svedman in Journal of Diabetes Science and Technology
