# Supplementary material for: A Cross-Sectional Study Demonstrating a High Prevalence of Skin Rash to Diabetes Medical Devices: An Underestimated Problem
Source: J Diabetes Sci Technol. 2025 May 7:19322968251336261. Online ahead of print. doi: 10.1177/19322968251336261 (PMC12058707; doi:10.1177/19322968251336261)
Supplement: sj-docx-1-dst-10.1177_19322968251336261 – Supplemental material for A Cross-Sectional Study Demonstrating a High Prevalence of Skin Rash to Diabetes Medical Devices: An Underestimated Problem [file sj-docx-1-dst-10.1177_19322968251336261.docx]

**Supplementary tables and figures**

**Supplementary table 1. Comparison of the proportion of respondents that have used different diabetes medical devices (diabetes MDs) stratified on age^a^.**

|  | **Use of MDs** | | | | | |
| --- | --- | --- | --- | --- | --- | --- |
| **Age group (years)** | **18-49** | | | **≥50** | | |
| **Different MDs**^b^ | **Respondents** |  |  | **Respondents** |  |  |
| **CGMs** |  |  |  |  |  |  |
| FreeStyle Libre | 273 | 256 | 93.8 | 290 | 270 | 93.1 |
| FreeStyle Libre 2 | 270 | 87 | 32.2 | 289 | 89 | 30.8 |
| Guardian sensor 3 | 269 | 62 | 23.0 | 289 | 24 | 8.3 |
| Dexcom G6 | 271 | 58 | 21.4 | 288 | 25 | 8.7 |
| Enlite | 265 | 20 | 7.5 | 279 | 16 | 5.7 |
| Dexcom G5 | 270 | 16 | 5.9 | 289 | 13 | 4.5 |
| Dexcom G4 | 269 | 12 | 4.5 | 287 | 10 | 3.5 |
| Guardian sensor 4 | 270 | 5 | 1.9 | 284 | 6 | 2.1 |
| Eversense | 265 | 3 | 1.1 | 280 | 2 | 0.7 |
| A6 Medtrum | 267 | 0 | 0 | 279 | 1 | 0.4 |
| **CSIIs** |  |  |  |  |  |  |
| MiniMed^c^ | 146 | 104 | 71.2 | 68 | 51 | 75.0 |
| Omnipod | 146 | 37 | 25.3 | 70 | 10 | 14.3 |
| Tandem t:slim X2 | 146 | 24 | 16.4 | 70 | 8 | 11.4 |
| Animas CSII | 146 | 6 | 4.1 | 70 | 6 | 8.6 |
| My life YpsoPump | 146 | 1 | 0.7 | 70 | 4 | 5.7 |

^a^Same inclusion criteria as in table 3.

^b^MDs with <50 users are shown in grey.

^c^Any version of the CSII.

Abbreviations: CGMs, continuous glucose monitors; CSII, continuous subcutaneous insulin infusion; MDs, medical devices.

**Supplementary table 2. Numbers of different diabetes medical devices (diabetes MDs^a^) used in different subgroups^b^.**

|  |  | **Number of diabetes MDs used** | | | | | | | | | | |
| --- | --- | --- | --- | --- | --- | --- | --- | --- | --- | --- | --- | --- |
|  | All | 1 | | 2 | | 3 | | 4 | | ≥5 | | P value^c^ |
| **Age group** |  | N | % | N | % | N | % | N | % | N | % | <0.001 |
| 18-49 years | 275 | 64 | 23.3 | 83 | 30.2 | 69 | 25.1 | 36 | 13.1 | 23 | 8.4 |  |
| ≥50 years | 295 | 151 | 51.2 | 79 | 26.8 | 32 | 10.8 | 24 | 8.1 | 9 | 3.1 |  |
| **Gender** |  |  |  |  |  |  |  |  |  |  |  | <0.001 |
| Male | 299 | 125 | 41.8 | 97 | 32.4 | 46 | 15.4 | 23 | 7.7 | 8 | 2.7 |  |
| Female | 271 | 90 | 33.2 | 65 | 24.0 | 55 | 20.3 | 37 | 13.7 | 24 | 8.9 |  |
| **Childhood atopic dermatitis (AD)** |  |  |  |  |  |  |  |  |  |  |  | 0.014 |
| Yes | 127 | 38 | 29.9 | 39 | 30.7 | 21 | 16.5 | 18 | 14.2 | 11 | 8.7 |  |
| No | 443 | 177 | 40.0 | 123 | 27.8 | 80 | 18.1 | 42 | 9.5 | 21 | 4.7 |  |

^a^Continuous glucose monitors and continuous subcutaneous insulin infusion devices

^b^Same inclusion criteria as in table 3.

^c^P-value for trend (linear by linear association).

**Supplementary figures**

**Supplementary figure 1.**

^a^P-value for trend (linear-by-linear association): p<0.001.
